# Host metabolite producing endophytic fungi isolated from *Hypericum perforatum*

**DOI:** 10.1371/journal.pone.0217060

**Published:** 2019-05-21

**Authors:** Aruna Vigneshwari, Dávid Rakk, Anikó Németh, Sándor Kocsubé, Noémi Kiss, Dezső Csupor, Tamás Papp, Biljana Škrbić, Csaba Vágvölgyi, András Szekeres

**Affiliations:** 1 Department of Microbiology, Faculty of Science and Informatics, University of Szeged, Szeged, Hungary; 2 Doctoral School of Biology, Faculty of Science and Informatics, University of Szeged, Szeged, Szeged, Hungary; 3 Botanical Garden, University of Szeged, Szeged, Szeged, Hungary; 4 Department of Pharmacognosy, Faculty of Pharmacy, University of Szeged, Szeged, Hungary; 5 MTA-SZTE Fungal Pathogenicity Mechanisms Research Group, Hungarian Academy of Sciences—University of Szeged, Szeged, Hungary; 6 Faculty of Technology, University of Novi Sad, Novi Sad, Serbia; 7 Interdisciplinary Centre of Natural Products, University of Szeged, Szeged, Hungary; Tallinn University of Technology, ESTONIA

## Abstract

In the present study, endophytic fungi have been isolated from various parts of the medicinal herb *Hypericum perforatum* (St. John’s Wort), which is known as a source of medically important metabolites. The isolated strains were cultured in liquid media and their ability to synthesize hypericin, the secondary metabolite of the host and its suspected precursor, emodin was tested analyzing the extracts of the fermentation broth and the mycelia. The HPLC-UV analysis of the chloroform/methanol extracts of the mycelia revealed that three isolates were able to produce emodin (SZMC 23771, 19.9 ng/mg; SZMC 23772, 20.8 ng/mg; SZMC 23769, 427.9 ng/mg) and one of them also could synthesize hypericin (SZMC 23769, 320.4 ng/mg). These results were also confirmed via UHPLC-HRMS technique both in full scan and MS/MS mode. The strains producing only emodin belong to the section *Alternata* of the genus *Alternaria*, while the isolate producing both metabolites was identified as *Epicoccum nigrum*. The mycelial extracts of *E*. *nigrum* and the *Alternaria* sp. SZMC 23772 showed higher inhibitory activities in the antimicrobial tests against the six selected bacteria compared to the hypericin and emodin standards in the applied concentration (100 μg/mL), while in case of the *Alternaria* sp. SZMC 23771 lower inhibition activities were observed on *Staphylococcus aureus* and *Streptomyces albus* than the pure compounds.

## Introduction

*Hypericum perforatum* L. (common St. John’s wort) is a widely distributed medicinal herb, which has been used over the past 2000 years for diverse healing purposes [1]. The genus *Hypericum* is belonging to the *Hypericaceae* family involving almost five hundred species [2]. Most of them can synthesize metabolites possessing antioxidant [3], anticancer [4], antidepressant [1], antiviral [5], antifungal and antibacterial effects [6]. The key component of these biological activities is the naphthodianthrone derivative hypericin, which can be a potential lead molecule for future therapeutics [7]. The biosynthesis of hypericin has not yet been clarified experimentally, but it is presumed to follow the polyketide pathway and to start with the condensation of seven malonyl- and one acetyl-CoA molecules. After that, the resulted octaketide chain undergoes both cyclization and decarboxylation reactions to form emodin anthrone, which is oxidized to emodin probably by the enzyme emodinanthrone-oxygenase and then, a condensation reaction yields a dianthrone leading to the formation of protohypericin and finally of hypericin [7]. This biosynthetic pathway is generally accepted and some genes encoding enzymes potentially involved in the biosynthesis have been already analyzed by next generation sequencing technology [8]. The spatial distribution of the chemical components of the biosynthetic pathway *in planta* were determined with desorption electrospray ionization mass spectrometry imaging (DESI-MSI) [9] and matrix free UV-laser desorption/ionization mass spectrometric imaging (LDI-IMS) [10] as well as by matrix-assisted laser desorption/ionization high-resolution mass spectrometry (HRMS) techniques [11]. In these studies, hypericin was found to be localized in the dark glands on leaves of *H*. *perforatum*, but the proposed precursor, emodin anthrone, could not be visualized. Due to its high reactivity, emodin anthrone can be instantaneously converted to emodin by oxidation. However, the other main proposed precursor, emodin was not only accumulated in the dark glands, but was also detected outside the glands in significant amounts suggesting that the presumed site of hypericin biosynthesis is in the cells adjacent to these gland structures from emodin [11]. Besides *Hypericum* sp., hypericin has also been found in some species of the basidiomycete genus *Dermocybe* [12,13] and in an undetermined filamentous fungus, which was isolated as an endophyte of *H*. *perforatum* [6].

Endophytes are the microorganisms residing in the internal tissues of the plants in a symbiotic relationship without any apparent symptoms of infections [14]. This special ecological niche together with the continual metabolic interactions between the fungus and the plant seems to serve as a strong evolutionary pressure for the endophytes to synthesize secondary metabolites [15], which may improve the fitness of the host plant and its resistance to various pests [16]. Furthermore, it has also been discovered that the produced metabolites are occasionally the same as those, which have originally been isolated and known from the plant hosts [17]. This observation promoted the isolation of several fungal endophytes producing important medicinal agents including digoxin, ginkgolides and podophyllotoxin originally described from *Digitalis lanata* [18], *Ginkgo biloba* [19] and *Juniperus communis* [20], respectively, as well as vincamine and vinpocetine isolated firstly from *Vinca minor* [21]. An endophytic strain of *Thielavia subthermofila* isolated from *H*. *perforatum* was capable to produce both hypericin and emodin in submerged axenic culture *in vitro* [6]. Furthermore, in contrast to the plant host, the fungus produced these compounds independently of the illumination conditions indicating that the biosynthetic pathway might be differently regulated in the fungus and the host plant [22].

Although it is possible to chemically synthesize hypericin and emodin [23], its major source is the St. John’s wort [7]. In modern medicine, the aerial parts of the plant (*Hyperici herba*) are applied to prepare extracts, which contain the active substances in a complex mixture [24]. Endophytic microorganisms can serve as cost-effective alternative sources of these plant metabolites. However, development of such applications requires an intensive seeking for new producer strains. Therefore, the present study focused on the isolation and identification of endophytic fungi, which can produce hypericin and emodin, the host metabolites of *H*. *perforatum*.

## Materials and methods

### Isolation of endophytes from *Hypericum perforatum*

Plant specimens of *Hypericum perforatum* were sampled at the Botanical garden of University of Szeged (N46.235, E20.159). Each collected specimen was placed in a sealed plastic bag and was labelled with the number and date of collection and stored at 4°C until processing. Isolation of endophytic fungi from plant parts was done according to the method described by Garyali [25] with minor modifications. The plant materials were rinsed in running tap water to remove dust and debris and the specimens were cut into small segments of about 0.5 to 1 cm in length using a sterile blade. The plant segments were surface sterilized to kill the epiphytic microorganisms by sequentially immersing the plant material in 70% ethanol for 60 s, washing with sterile distilled water and then, steeping in 0.01% mercuric chloride (VWR) for 30 sec. Finally, the specimens were washed again with sterile distilled water 2–3 times and then allowed to dry on a sterile blotting paper. Each segment was placed onto the surface of potato dextrose agar (PDA; VWR) medium supplemented with ampicillin (50 μg/mL) in a Petri dish. All plates were incubated at 25°C for 5–10 days and were checked daily for the growth of fungal colonies. Pure isolates were obtained by picking up individual colonies from the plates and transferring them onto a fresh PDA medium where they were incubated at 25°C for 10 days. Each fungal culture was checked again for purity and transferred separately to PDA slants and maintained at 4°C and this generation (3th) of the isolates were deposited into the Szeged Microbiological Collection (SZMC, Hungary, http://szmc.hu/). For the investigations of the metabolite production, the subcultures of these deposited isolates were applied, which was the fourth subcultivation of the endophytes.

### Molecular identification

For DNA isolation, fungal isolates were grown in potato dextrose broth (PDB; VWR) for 7 days at 25°C. Isolation of the genomic DNA from the mycelia was performed using E.Z.N.A. Fungal DNA Kit (Omega Bio-tek) according to the manufacturer’s instructions. The internal transcribed spacer (ITS) region of the rDNA was amplified using the primers ITS1 and ITS4as described previously [26]. Sequencing of the amplified DNA fragments was performed on an ABI 373A DNA sequencer (Applied Biosystems Inc., USA) using dye dideoxy terminator reaction chemistry. The sequences were first analyzed by BLAST similarity search at the website of the National Center for Biotechnology Information (http://www.ncbi.nlm.nih.gov/BLAST) and the species were identified based on their identity values (>97%). Identification of the SZMC 23773 strain was also reinforced using the online software *Trich*Okey 2.0 (www.isth.info) [27].

### Phylogenetic analysis of the producer strains

In the case of *Alternaria* strains, the ITS sequences of the producer strains were aligned to those of the ex-type and representative strains [28,29] using the CLUSTAL_X software [30]. The species involved and the GenBank accession numbers of their sequences are given in S1 Table. The ITS sequence of the strain CBS 191.86 of *Stemphylium herbarum* (KC584239) was used as the outgroup. The phylogenetic tree was constructed with the neighbor-joining method using 1,000 bootstrap replicates [31]. The evolutionary distances were computed using the p-distance method [32] and were given in the units of the number of base differences per site. All ambiguous positions were removed for each sequence pair and there were 368 positions in the final dataset. The phylogenetic analyses and the tree construction were conducted in MEGA7 [33].

### Preparation of the metabolite extracts

Isolated endophytic fungi were cultured for 7 days at 25°C in 50 ml PDB medium. The extraction was carried out according to Kusari et al. [6] with minor modifications. The mycelia were separated from the broth by filtration through a cheese cloth and overnight dried in an oven until constant weight, which was determined. Then 25 mL distilled water was added to the dry material, which was then sonicated for 20 min after the addition of an aliquot of liquid nitrogen to maintain the chilled condition. This aqueous solution was extracted then three times, firstly, with 25 mL ethyl acetate and then, with 25 mL chloroform-methanol (4:1), and the extracts obtained with the same solvent were pooled. Fifty mL of the ferment broth was also extracted three times sequentially with 50–50 mL of ethyl-acetate and chloroform-methanol (4:1), respectively, and the extracts were also pooled. The organic solvents from each pooled extract were removed by a rotary evaporator (IKA HB10 basic, VWR) in vacuum at 30°C. The resulted four dry samples per each isolate were stored at -20°C and resuspended in 1 mL of HPLC grade methanol (VWR) prior to use.

### Antimicrobial assays

Antimicrobial effect of pure hypericin and emodin (Sigma) and the methanolic solutions of the samples extracted from the mycelia and the ferment broths were tested using the microdilution method against the bacterial strains *Escherichia coli* (SZMC 0582), *Pseudomonas aueroginosa* (SZMC 21886), *Staphylococcus aureus* (SZMC 14532), *Bacillus subtilis* (SZMC 14624) *Micrococcus luteus* (SZMC 6207) and *Streptomyces albus* (SZMC 0282) according to the M07-A10 CLSI guideline [34]. Suspensions of the bacteria were prepared from overnight cultures cultivated in nutrient broth (NB, 1 g/L peptone, 15 g/L sodium chloride, 6 g/L yeast extract) at 37°C and the concentrations of the suspensions were adjusted to 4 x 10^5^ cells/mL. The extracts resuspended in methanol were diluted with water to reach the methanol content up to 10%. The 96-well plates were prepared by dispensing into each well 100 μL of NB containing the bacterial cells and 100 μL of extracts and incubated for 24 h at 37°C. The mixture of 100 μL NB and 100 μL extracts were used as the blank sample for the background correction, while 100 μL of bacterial cultures supplemented with 100 μL of 10% methanol or 100 μg/mL ampicillin (Sigma) solution were applied as the positive and the negative controls, respectively. The pure compounds were applied in a concentration of 100 μg/mL. Absorbances were measured at 620 nm after 1 and 24 hours of incubation and inhibition (%) was calculated as the percentage of the positive control after the blank correction.

### HPLC-UV analysis

The applied analytical method was based on the description of Li et al. [35] with slight modifications. The extracts were analyzed by the modular HPLC system (Shimadzu, Japan) equipped with a fluorescence detector, which was controlled by ClassVP 6.2 software. The peaks were detected by an UV detector at a wavelength of 436 nm. The mobile phase consisted of water containing 20% methanol (A) and acetonitrile containing 10% methanol (B) and both were supplemented with 0.5% trifluoroacetic acid (Sigma). Separations were performed on a Gemini 250 × 4.6 mm, 5 μm reversed phase column (Phenomenex, Torrance, CA) coupled with Phenomenex C18 guard column with a flow rate of 1 mL/min using a gradient program started with 10% B, and reached to 70% B until 10 min, to 90% until 15 min and to 25 min until 100%, which was kept until 60 min and reduced to initial eluent ratio and held to pressure stabilization. The injection volume was 5 μL. The calibration was done with serial dilution of hypericin and emodin standards (Sigma) in the range of 250 μg/mL to 7.8 μg/mL based on the retention times of hypericin (32.8 min) and emodin (16.9 min). The quantity of hypericin and emodin present in the samples were quantified using the equations y = 0.000142788 x—5.07 and y = 0.0000808111 x—4.66, respectively, while the r values were 0.998 and 0.999 for hypericin and emodin, respectively.

### HPLC-HRMS and HRMS/MS analysis

The identity of hypericin and emodin were confirmed by a Thermo Q Exactive Plus high-resolution mass spectrometer (Thermo Scientific), which was equipped with a Waters UPLC I-Class System (Waters) consisting of a binary pump, a column manager and a fixed loop auto sampler. The separations were performed by using a Phenomenex Kinetex XB-C18 column (2.6 μm, 2.1 × 50 mm, 100 Å) (Torrance) with water (A) and acetonitrile (B) eluents containing 0.1% formic acid with a flow rate of 0.5 mL/min at 40°C. Samples and standards were analyzed using a gradient program as follows: from 5% B linear gradient to 95% B over 10 min and after 95% B isocratic for 2.5 min, the system returned to its initial condition (5% B) within 0.1 min and was equilibrated for 2.4 min. The spectrometer was operated in data dependent MS^2^ mode with negative electrospray ionization (ESI) (number of precursors: Top 5; scan range 100–1500; dynamic exclusion: 10 sec; 1 exclude isotopes: on; stepped NCE: 30, 50, 80) with nominal mass resolving power of 60 000 at *m/z* 200 with a scan rate of 1 Hz with automatic gain control to provide high-accuracy mass measurements within 2 ppm. Nitrogen was used as sheath gas, and as the collision gas. The source parameters were the followings: spray voltage (-): 2500.00, capillary temperature (-): 300.00, sheath gas (-): 55.00, aux. gas (-): 15.00, spare gas (-): 5.00, max spray current (-): 100.00, probe heater temp. (-): 450.00, S-lens RF level: 50.00.

### Statistical analysis

The statistical analysis was performed using the GraphPad Prism version 7.0 for Windows (GraphPad Software). To compare the inhibition effects of hypericin, emodin and the fungal extracts on the bacterial strains, the one-way analysis of variance (ANOVA) was used and p<0.05 was accepted as statistically significant.

## Result

### Identification of the endophytic fungi isolated from *H*. *perforatum*

The *H*. *perforatum* plants were collected from the Botanical Garden of the University of Szeged in autumn. The leaf, stem, root and flower parts were separated, and these parts were examined for their fungal endophyte content. Altogether 48 parts were tested involving 12–12 leaf, stem, root and flower cuttings, respectively. Then due to the intensive surface sterilization procedure, eight fungal strains were isolated from the samples after a 7-days incubation (Table 1). Three strains were isolated from the leaves, two fungi from the stems and the flowers and one strain from the root. The isolated fungi belonged to the genera *Alternaria* (six strains), *Epicoccum* (one strain) and *Trichoderma* (one strain) (Table 1). By the NCBI BLAST search using the ITS sequence, the *Trichoderma* strain proved to be *T*. *harzianum*. This result was additionally confirmed by the *Trich*Okey barcode identification system based on the five specific hallmarks found in the sequence.

**Table 1:** List of the isolated and identified endophytes.

Genbank accession number	Species^a^	Collection code	Plant part
KY613791	*Epicoccum nigrum*	SZMC 23769	Flower
KY613792	*Alternaria sp*.	SZMC 23770	Stem
KY613793	*Alternaria sp*.	SZMC 23775	Flower
KY613794	*Alternaria sp*.	SZMC 23776	Stem
KY613795	*Alternaria sp*.	SZMC 23774	Root
KY613796	*Alternaria sp*.	SZMC 23771	Leaf
KY613797	*Alternaria sp*.	SZMC 23772	Leaf
KY613798	*Trichoderma harzianum*	SZMC 23773	Leaf

^a^Identity based on the comparison of the first blast hit at 29 May 2018.

**Fig 1:**
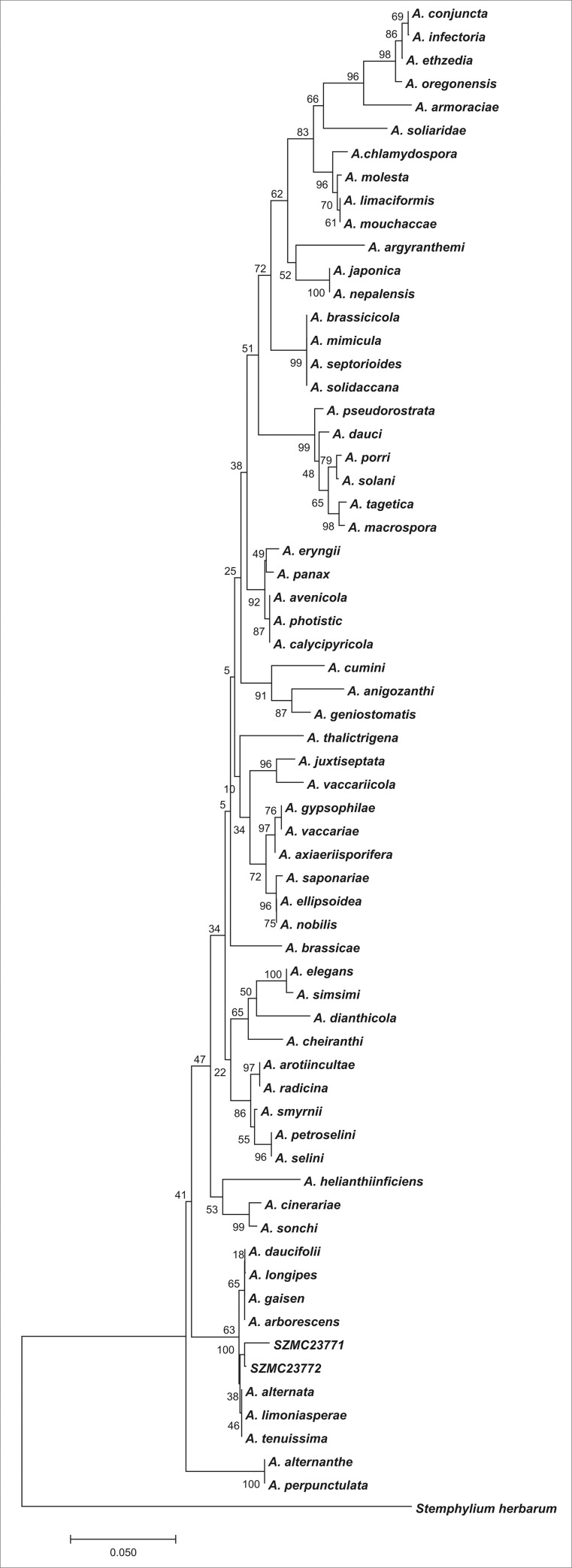
Phylogenetic analysis of the *Alternaria* strains proved to be the producer of emodin. The tree was rooted to *Stemphylium herbarum*. The percentage of replicate trees, in which the associated taxa clustered together in the bootstrap test (1000 replicates), were positioned next to the branches [36].

According to the NCBI hits, both emodin producer strains (SZMC 23771 and 23772) strains were identified as *Alternaria* sp. (Table 1). Based on the phylogenetic analysis of the ITS sequences of these two strains as well as those of the ex-type and representative strains of the species available in the GenBank, the isolate belongs to the section *Alternata* (Fig 1).

The isolate SZMC 23769, which produces hypericin and emodin, was identified as *Epicoccum nigrum* via the BLAST search of the NCBI GenBank (Table 1).

### Detection of hypericin and emodin in the isolated endophytes

Ethyl-acetate and chloroform-methanol extracts of the mycelia and the ferment broths of the isolated endophytes were examined for the presence of hypericin and emodin by an HPLC-UV analysis.

**Fig 2:**
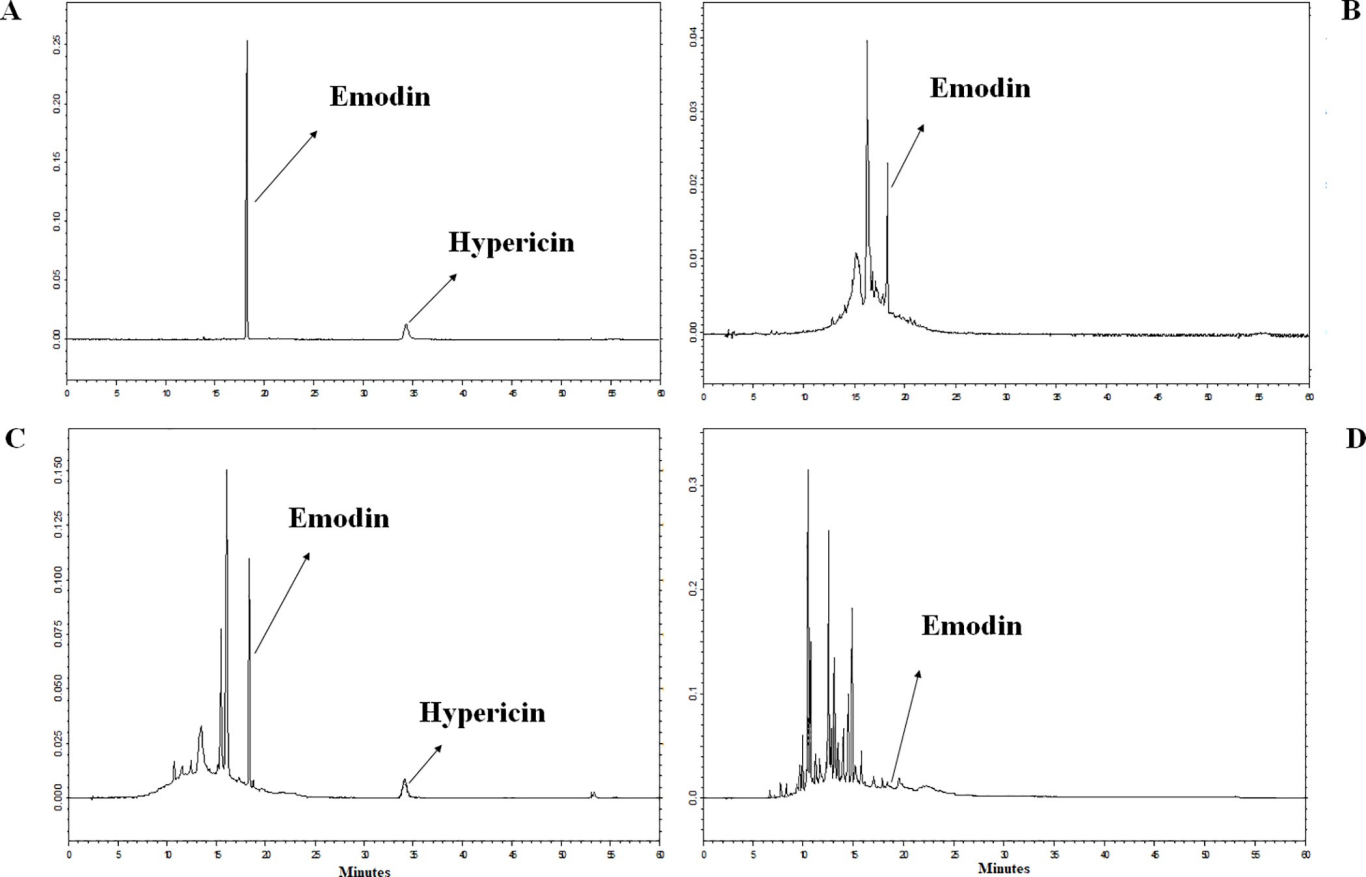
HPLC-UV chromatogram of the producer strains. The standard mixture of 1 and 2 (A) as well as the mycelial extract of SZMC23771 (B), SZMC23769 (C) and SZMC23772 (D) extracted with chloroform-methanol.

Altogether, 32 extracts were checked for the presence of the metabolites. In certain cases, the observed peaks detected in the extracts fitted well to the retention time of the standard compounds (Fig 2). None of the ethyl-acetate extracts contained the examined analytes in measurable amount. At the same time, emodin could be detected in the chloroform-methanol extracts of the SZMC 23772 and SZMC 23771 mycelia and both hypericin and emodin were found in the mycelial extract of SZMC 23769. It is important to emphasis that the metabolites were only observed in the mycelial extracts suggesting that the compounds may be produced either intracellularly or associated to the surface of the fungal cell wall. Both *Alternaria* strains (i.e. SZMC 23771 and SZMC 23772) produced emodin in similar amount. Compared to them, *E*. *nigrum* contained more than 20 times more of this compound (i.e. over 2 μg/mL broth cultured within the applied conditions). The hypericin yield of SZMC 23769 was approximately three quarters of that of the emodin produced by the same strain (Table 2).

**Table 2:** List of the strains producing hypericin and emodin and their detected amounts in the ethyl-acetate extracts of the mycelia.

	Emodin (ng/mg)	Hypericin (ng/mg)
Strain number	referred to the mycelial weight	
**SZMC 23771**	19.9 (4.1)	BDL
**SZMC 23772**	20.8 (20.5)	BDL
**SZMC 23769**	427.9 (37.4)	320.4 (25.0)

The relative standard deviations (%) are in brackets, while BDL mean below detection limit.

### Confirmation of the identity of the detected metabolites by mass spectrometry

**Fig 3:**
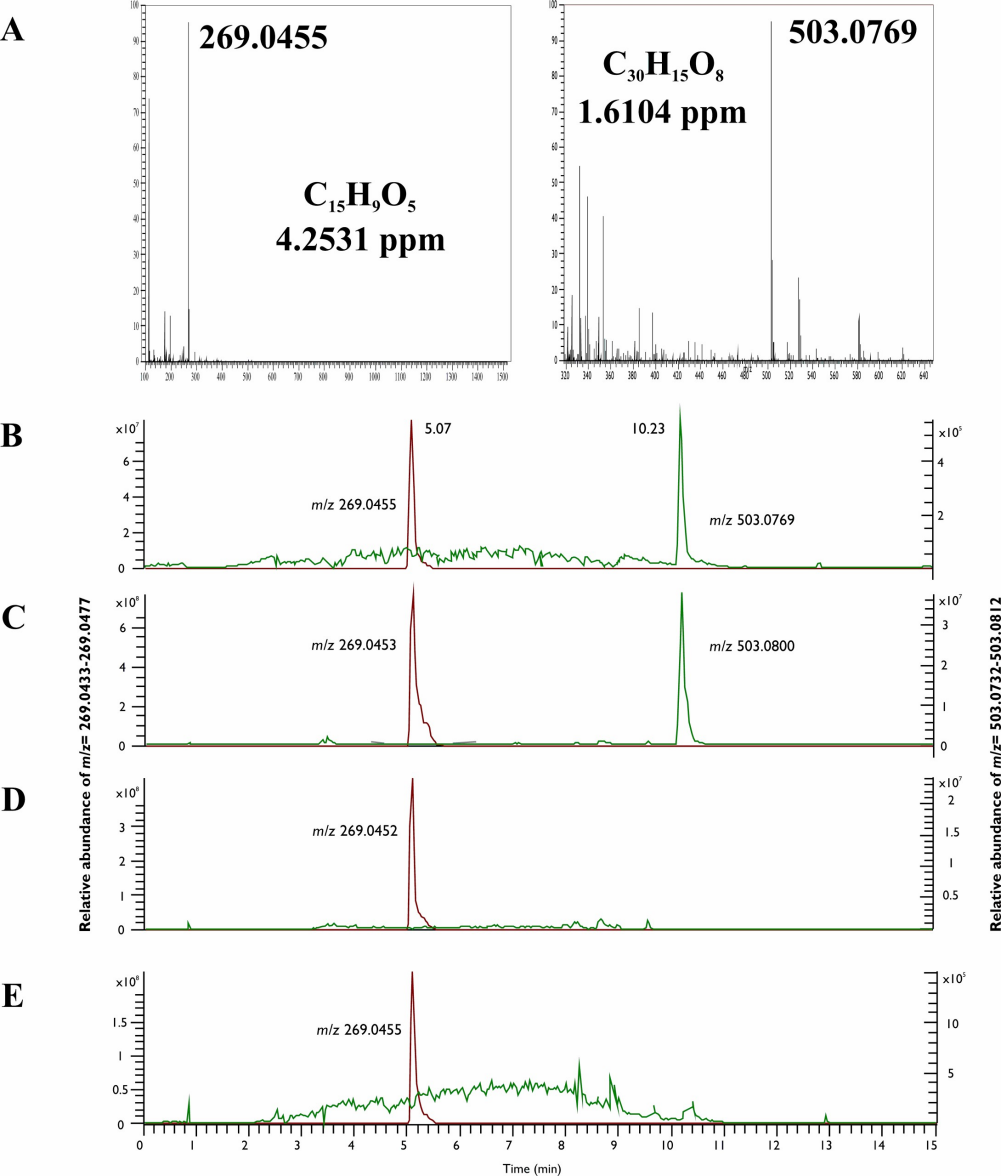
Full scan MS examinations of the hypericin and emodin production of the endophytes. The full scan MS spectra of emodin (C_15_H_9_O_5_) and hypericin (C_30_H_15_O_8_) (A) and chromatograms of the standard hypericin and emodin (B), as well as the mycelial extract of SZMC 23769 (C), SZMC 23771 (D) and SZMC 23772 (E) extracted at the *m/z* values of hypericin (*m/z* 503.0732–503.0812) and emodin (*m/z* 269.0433–269.0477).

**Fig 4:**
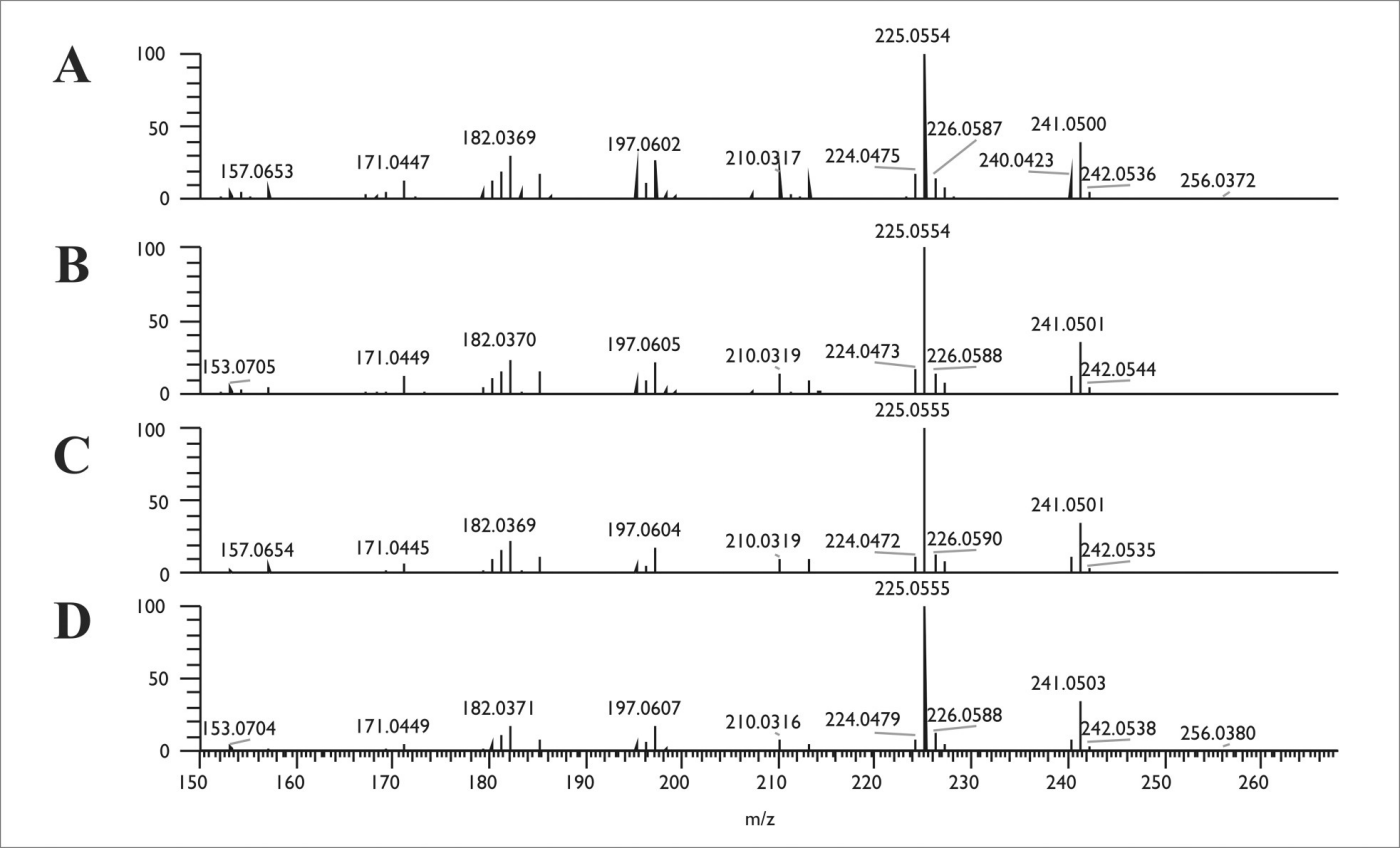
The MS2 examinations of the emodin production of the endophytes. The MS2 spectra of emodin (C_15_H_9_O_5_) standard (A) and the mycelial extract of SZMC 23769 (B), SZMC 23771 (C) and SZMC 23772 (D) recorded at the retention time of 5.07 min.

**Fig 5:**
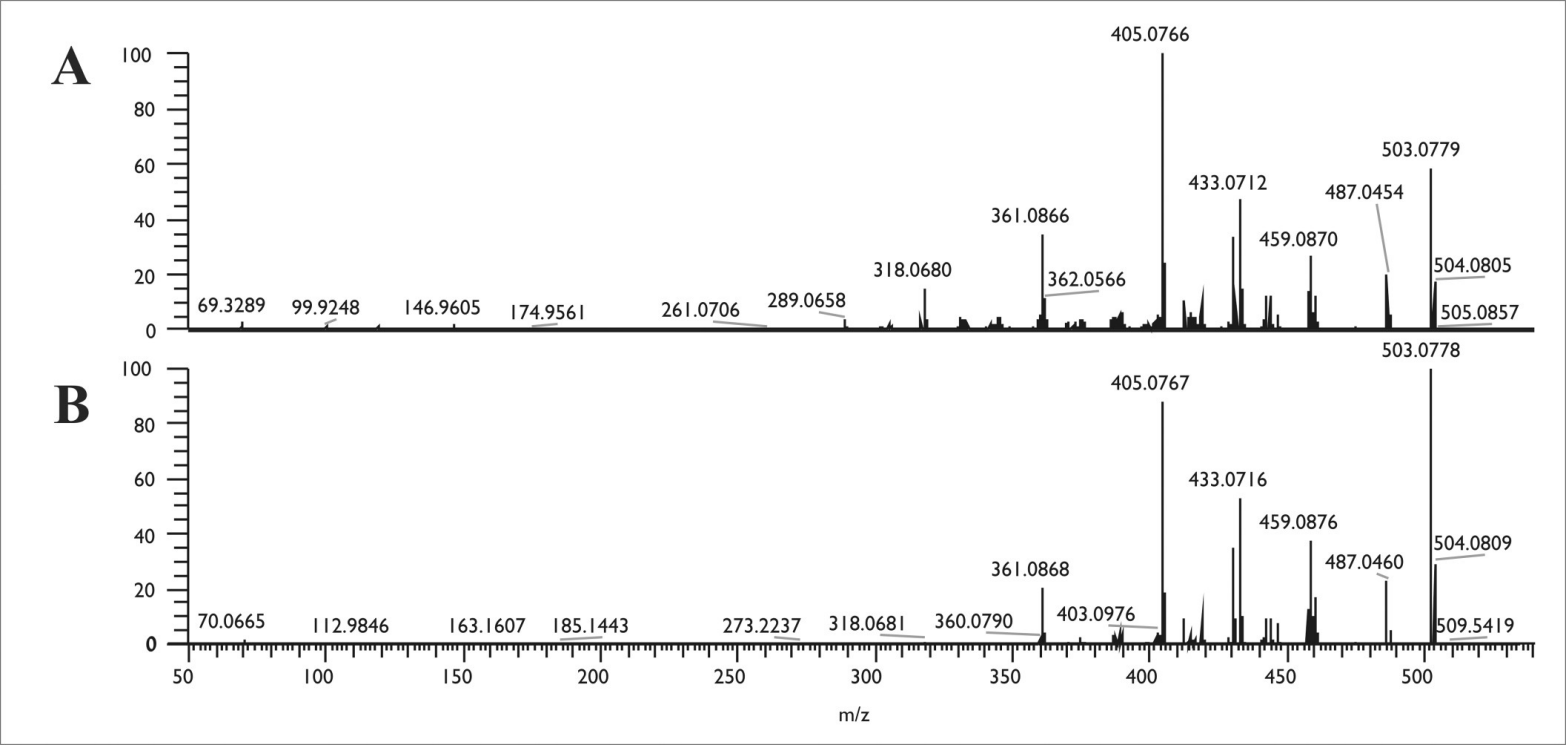
The MS2 examinations of the hypericin production of the endophytes. The MS2 spectra of hypericin (C_30_H_15_O_8_) standard (A) and the mycelial extract of SZMC 23769 (B) recorded at the retention time of 10.23 min.

Confirmation of the HPLC-UV detection of hypericin and emodin was performed by comparing the extracts with authentic reference standards using LC-HRMS and LC-HRMS/MS techniques. Good ionization properties were obtained in negative ESI mode for both hypericin and emodin during the MS optimization procedures, which was used later to record the high-resolution full scan ESI-MS spectra of both the standard and the fungal compounds. The retention times of the suspected hypericin and emodin peaks were equivalent with the standard compounds as in the case of HPLC-UV measurement (Fig 3). The molecular formulas of the compounds were determined by the high mass resolution of the applied instrument, which proved to be C_15_H_10_O_5_ ([M-H]^-^, 269.0456) for emodin in the case of each producer and resulted to C_30_H_16_O_8_ ([M-H]^-^, 503.0770) for hypericin in the case of SZMC 23769. Moreover, the full scan spectra were identical to the data obtained for the authentic standards (Fig 3). Within data dependent MS2 mode, the resulted patterns of the fragments after the collision of the above-mentioned ions as precursor ions in the HCD cell also corresponded to the hypericin and emodin standards (Figs 4 and 5).

### Antibacterial activity of hypericin and emodin

**Fig 6:**
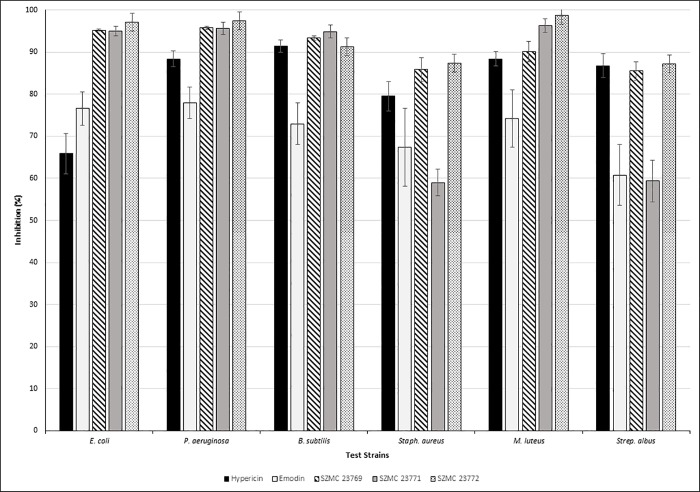
Antibacterial activities of the standard solutions and the three selected fungal extracts against the tested bacteria. Significant differences were observed in all cases between the inhibitory values of the sample extracts and the hypericin solution (ρ < 0.05) as well as between the inhibitory values of the sample extracts and emodin solution (*ρ* < 0.05).

One hundred μg/mL concentration of the reference standards of the two metabolites were tested against six bacteria. Hypericin and emodin showed moderate to high inhibitions against each bacterium in the ranges of 65% - 92% and 60% - 78%, respectively. Hypericin exerted the highest antimicrobial activity on *B*. *subtilis* and the lowest on *E*. *coli*. Emodin had the highest inhibitory effect on *P*. *aeruginosa* and the lowest on *Strep*. *albus*. It could also be observed that the antibacterial effect of hypericin was generally higher than that of emodin, except for the effects on *E*. *coli* (Fig 6).

In general, mycelial extracts of all isolates showed higher inhibitory activity on the bacteria than the broth extracts using either ethyl-acetate or chloroform-methanol solvents for the pretreatment (data not showed). Data of the extracts containing the examined metabolites are presented in Fig 6. Chloroform-methanol extracts of the mycelia of *E*. *nigrum* SZMC 23769 and the *Alternaria* sp. SZMC 23772 showed higher activities against each bacterium than the hypericin and emodin standards in the applied concentration (100 μg/mL), while in case of the *Alternaria* sp. SZMC 23771, mycelial extracts displayed lower inhibition activities on the *Staphylococcus* and *Streptomyces* species than the standards. Referred to the dry weight (DW), concentrations of emodin in the ethyl-acetate extracts of SZMC 23771 (DW: 220.5 mg) and SZMC 23772 (DW: 183.9 mg) were 4.4 μg/mL and 3.8 μg/mL, respectively. Thus, the observed powerful antibacterial activities can be attributable not solely to the produced emodin but also to the presence of other components in the extract. The concentration of hypericin and emodin in the mycelial extract of *E*. *nigrum* (DW: 273.6 mg) were 117.1 μg/mL and 87.7 μg/mL, respectively. These concentrations are similar to the applied concentrations of the pure standards, but their observed effects were higher. However, while the standards were tested separately, both compounds were present in the extract, thus they could enhance the effects of each other.

## Discussion

The present study was based on the observation that certain endophytes are able to produce the same metabolites as their plant hosts and thus, they can serve as novel microbial sources of bioactive plant metabolites [37,38]. Hypericin is one of the medicinally important polyphenolic compounds as it has been proven to have antidepressant, antitumor and antiviral properties and it is also used in photodynamic therapy for the detection and treatment of tumor cells [7]. Furthermore, only a single case has been reported until today when the plant metabolite, hypericin could be produced by an endophyte [6,22]. In that report, hypericin occurred together with its biosynthetic precursor, emodin. In our study, a seeking for other possible fungal producers was performed by sampling of plant material, isolation of fungal strains and analytical examinations.

In our study, the isolates producing hypericin or emodin, were identified as members of *Alternaria* and *Epicoccum* genus. The *Alternaria* isolates were identified only at the section level, because members of the *A*. *alternata* species group including *A*. *tenuissima*, *A*. *arborescens*, and *A*. *alternata* cannot be discerned based either on the ITS sequence or on multigene approach [39,40]. Based on the recent classification of the *Alternaria* genus, the support values (Bayesian posterior probabilities, RAxML bootstrap) of the section *Alternata* were sufficiently high for the discrimination [28]. The corresponding clade involves species that are commonly referred in the literature as small spored *Alternaria* [41]. There are about 300 known *Alternaria* species and they have been clustered in several species-groups according to phylogenetic studies [28,41]. Numerous morphological species have been described within the genus representing same species or discrete evolutionary taxa [42], in which eight phylogenetic lineages were identified assigning them the taxonomic rank of section [43], which was later re-classified into 23 [28] and recently into 27 sections [41]. It has already proven that *Alternaria* species associated to plants were able to produce the host metabolites methyl-eugenol [44], capsaicin [45] and paclitaxel [46] but, according to our knowledge, their emodin production ability has not yet been reported. It is interesting that emodin was previously found to be an efficient antifungal toxin isolated from *Rhamnus triquetra* bark showing strong inhibition of the spore germination of 17 tested fungal species including seven *Alternaria* spp. [47].

*E*. *nigrum* is anamorphic ascomycete distributed worldwide and considered to be a saprophytic fungus, although it can also show an endophytic lifestyle [48,49] and can be frequently isolated from the inner tissues of various plants [50]. Association between the plants and the *Epicoccum* species may also lead to the production of plant metabolites by the fungi such as taxol [51] and other unique bioactive compounds [52] such as epicorazin A and B [53], epicoccin A, B and D [54], epicoccarine A and B, epipyridone [55], flavipin [56] and epirodins [57]. However, our study has demonstrated firstly their abilities to produce hypericin and emodin.

Emodin is a well-known active ingredient of medicinal herbs [58], however, it has originally been identified from *Cortinarius sanguineus* (formerly known as *Dermocybe sanguinens*) as a colored metabolite [59] and it was also detected in *Cladosporium fulvum* [60], *T*. *subthermophila* [22] and *Aspergillus* species including *A*. *wentii* [61] and *A*. *ochraceus* [62].

Data concerning the amount of hypericin and emodin produced by fungi are not available in the literature, except for *T*. *subthermophila* isolated from *H*. *perforatum* [6]. That fungus produced 0.35 ng/mg (dry weight of the mycelium) hypericin and 1.13 ng/mg (dry weight of the mycelium) + under shake flask condition [6]. In case of *H*. *perforatum*, the average amounts of hypericin and emodin were found to be 3330 ng/mg (dry weight) and 190 ng/mg (dry weight), which could significantly decrease during a cold acclimation period and remained unchanged after the exposure of plants to dehydration and exogenous abscisic acid treatment [63]. Furthermore, the amount varied within the taxonomical category [64], seasons of harvesting [65], different plant structures [66] and the ontogenetic phases [67]. It is interesting that the emodin content of fungi is higher than that of the hypericin, which is vice versa in plants. In our study, *E*. *nigrum* produced hypericin and emodin in higher quantities than *T*. *subthermophila* (Table 2). Although the amounts of both compounds were lower in *E*. *nigrum* than those described for *H*. *perforatum*, they proved to be higher than the quantities reported for other *Hypericum* species [63].

Although the hypericin and emodin available from plant origin in relatively high amounts, the microbiological sources could offer new perspectives for their industrial production in the future. The possible microbiological fermentation could provide stable, robust and reproducible yields from batch to batch due to the well controllable cultivation parameters.

## Supporting information

S1 Table List of the ex-type and reference strains as well as the outgroup strain [28] used to phylogenetic analysis of host metabolite producer *Alternaria* strains. (PDF)
