# Supplementary material for: Host metabolite producing endophytic fungi isolated from Hypericum perforatum
Source: PLoS One. 2019 May 21;14(5):e0217060. doi: 10.1371/journal.pone.0217060 (PMC6529008; doi:10.1371/journal.pone.0217060)
Supplement: S1 Table — (PDF) [file pone.0217060.s001.pdf]

S1 Table. List of the ex-type and reference strains as well as the outgroup strain [1] used to phylogenetic analysis of host metabolite producer *Alternaria* strains

Species name	Status[1]	Genbank (ITS)	Genbank (RPB2)	Genbank (TEF1)	Host / Substrate	Country
A. alternata	T	AF347031.1	KC584375	KC584634	*Arachis hypogaea*	India
A. anigozanthi	T	KC584180	KC584376	KC584635	*Anigozanthus* sp.	Australia
A. arborescens	T	AF347033	KC584377	KC584636	*Lycopersicon esculentum*	USA
A. argyranthemi	T	KC584181	KC584378	KC584637	*Argyranthemum* sp.	New Zealand
A. armoraciae	T	KC584182	KC584379	KC584099	*Armoracia rusticana*	New Zealand
A. avenicola	T	KC584183	KC584380	KC584639	*Avena* sp.	Norway
A. axiaeriisporifera	T	KC584184	KC584381	KC584640	*Gypsophila paniculata*	Zealand
A. brassicae	R	KC584185	KC584382	KC584641	*Brassica oleracea*	USA
A. brassicicola	R	JX499031	KC584383	KC584642	*Brassica oleracea*	USA
A. calycipyricola	T	KC584186	KC584384	KC584643	*Pyrus communis*	China
A. carotiincultae	T	KC584188	KC584386	KC584645	*Daucus carota*	USA
A. cheiranthi	R	AF229457	KC584387	KC584646	*Cheiranthus cheiri*	Italy
A. chlamydospora	T	KC584189	KC584388	KC584647	Soil	Egypt
A. cinerariae	R	KC584190	KC584389	KC584648	*Ligularia* sp.	USA
A. conjuncta	T	FJ266475	KC584390	KC584649	*Pastinaca sativa*	Switzerland
A. cumini	T	KC584191	KC584391	KC584650	*Cuminum cyminum*	India
A. dauci	R	KC584192	KC584392	KC584651	*Daucus carota*	USA
A. daucifolii	T	KC584193	KC584393	KC584652	*Daucus carota*	USA
A. dianthicola	R	KC584194	KC584394	KC584653	*Dianthus allwoodii*	New Zealand
A. elegans	T	KC584195	KC584395	KC584654	*Lycopersicon esculentum*	Burkina Faso
A. ellipsoidea	T	KC584196	KC584396	KC584655	*Dianthus barbatus*	USA
A. eryngii	R	JQ693661	KC584397	KC584656	*Eryngium* sp.	-
A. ethzedia	T	AF392987	KC584398	KC584657	*Brassica napus*	Switzerland
A. gaisen	R	KC584197	KC584399	KC584658	*Pyrus pyrifolia* cv. Nijiseiki	Japan
A. geniostomatis	T	KC584198	KC584400	KC584659	*Geniostoma* sp.	New Zealand

Species name	Status[1]	Genbank (ITS)	Genbank (RPB2)	Genbank (TEF1)	Host / Substrate	Country
A. helianthiinficiens	R	KC584200	KC584401	KC584660	*Helianthus annuus*	UK
A. gypsophilae	T	KC584199	KC584402	KC584661	*Gypsophila elegans*	–
A. infectoria	T	DQ323697	KC584404	KC584662	*Triticum aestivum*	UK
A. japonica	R	KC584201	KC584405	KC584663	*Brassica chinensis*	USA
A. juxtiseptata	T	KC584202	KC584406	KC584664	*Gypsophila paniculata*	Australia
A. limaciformis	T	KC584203	KC584407	KC584665	Soil	UK
A. limoniasperae	T	FJ266476	KC584408	KC584666	*Citrus jambhiri*	USA
A. longipes	R	AY278835	KC584409	KC584667	*Nicotiana tabacum*	USA
A. macrospora	T	KC584204	KC584410	KC584668	*Gossypium barbadense*	USA
A. mimicula	T	FJ266477	KC584411	KC584669	*Lycopersicon esculentum*	USA
A. molesta	T	KC584205	KC584412	KC584670	*Phocaena phocaena*	Denmark
A. mouchaccae	T	KC584206	KC584413	KC584671	Soil	Egypt
A. nepalensis	T	KC584207	KC584414	KC584672	*Brassica* sp.	Nepal
A. nobilis	R	KC584208	KC584415	KC584673	*Dianthus caryophyllus*	New Zealand
A. oregonensis	T	FJ266478	KC584416	KC584674	*Triticum aestivum*	USA
A. panax	R	KC584209	KC584417	KC584675	*Aralia racemosa*	USA
A. perpunctulata	T	KC584210	KC584418	KC584676	*Alternanthera philoxeroides*	USA
A. petroselini	T	KC584211	KC584419	KC584677	*Petroselinum sativum*	–
A. photistic	T	KC584212	KC584420	KC584678	*Digitalis purpurea*	UK
A. porri	R	DQ323700	KC584421	KC584679	*Allium cepa*	USA
A. pseudorostrata	T	JN383483	KC584422	KC584680	*Euphorbia pulcherrima*	USA
A. radicina	T	KC584213	KC584423	KC584681	*Daucus carota*	USA
A. saponariae	R	KC584215	KC584425	KC584683	*Saponaria officinalis*	USA
A. selini	T	AF229455	KC584426	KC584684	*Petroselinum crispum*	Saudi Arabia
A. septorioides	T	KC584216	KC584427	KC584685	*Reseda odorata*	Netherlands
A. simsimi	T	JF780937	KC584428	KC584686	*Sesamum indicum*	Argentina
A. smyrnii	R	AF229456	KC584429	KC584687	*Smyrnium olusatrum*	UK
A. solani	R	KC584217	KC584430	KC584688	*Solanum tuberosum*	USA
A. soliaridae	T	KC584218	KC584431	KC584689	Soil	USA

Species name	Status[1]	Genbank (ITS)	Genbank (RPB2)	Genbank (TEF1)	Host / Substrate	Country
A. solidaccana	T	KC584219	KC584432	KC584690	Soil	Bangladesh
A. sonchi	R	KC584220	KC584433	KC584691	*Sonchus asper*	Canada
A. tagetica	R	KC584221	KC584434	KC584692	*Tagetes erecta*	UK
A. tenuissima	R	AF347032	KC584435	KC584693	*Dianthus* sp.	UK
A. thalictrigena	T	EU040211	KC584436	KC584694	*Thalictrum* sp.	Germany
A. vaccariae	R	KC584223	KC584438	KC584696	*Vaccaria hispanica*	USA
A. vaccariicola	T	KC584224	KC584439	KC584697	*Vaccaria hispanica*	USA
Stemphylium herbarum	O	KC584239	KC584471	KC584373	*Medicago sativa*	India

[1]T: ex-type strain; R: representative strain; O: outgroup

1. Woudenberg JHC, Groenewald JZ, Binder M, Crous PW. *Alternaria* redefined. Stud Mycol. 2013;75: 171–212. doi:10.3114/sim0015
